# Synthesis and crystal structure of 1,1′-bis­{[4-(pyridin-2-yl)-1,2,3-triazol-1-yl]meth­yl}ferrocene, and its complexation with Cu^I^

**DOI:** 10.1107/S2056989020011901

**Published:** 2020-09-04

**Authors:** Uttam R. Pokharel, Aaron P. Naquin, Connor P. Brochon, Frank R. Fronczek

**Affiliations:** a Department of Chemistry & Physical Sciences, Nicholls State University, Thibodaux, Louisiana 70301, USA; b Department of Chemistry, Louisiana State University, Baton Rouge, Louisiana, 70803, USA

**Keywords:** 1,1′-bis­(pyridyl­triazoylmeth­yl)ferrocene, crystal structure, click reaction, tetra­dentate ligand, disubstituted ferrocene, copper(I) complex

## Abstract

The title compound, [Fe(C_13_H_11_N_4_)_2_], was synthesized starting from 1,1′-ferro­cene­di­carb­oxy­lic acid in a three-step reaction sequence. The di­carb­oxy­lic acid was reduced to 1,1′-ferrocenedi­methanol using LiAlH_4_ and subsequently converted to 1,1′-bis­(azido­meth­yl)ferrocene in the presence of NaN_3_. The diazide was treated with 2-ethynyl­pyridine under ‘click’ conditions to give the title compound in 75% yield. The Fe^II^ center lies on an inversion center in the crystal. The two pyridyl­triazole wings are oriented in an *anti* conformation and positioned *exo* from the Fe^II^ center. In the solid state, the mol­ecules inter­act by C—H⋯N, C—H⋯π, and π–π inter­actions. The complexation of the ligand with [Cu(CH_3_CN)_4_](PF_6_) gives a tetra­nuclear dimeric complex.

## Abstract 2

The ferrocene-bridged compound 1,1′-bis­(pyridyl­triazoylmeth­yl)ferrocene acquires an *anti* conformation in its solid state but forms a discrete tetra­nuclear [2 + 2] complex with [Cu(CH_3_CN)_4_](PF_6_) in solution.

## Chemical context   

Metal–organic supra­molecular chemistry is an emerging area in inorganic chemistry: the structurally challenging functional supra­molecules can be constructed from the self-assembly of multidentate organic ligands and transition-metal ions in relatively few synthetic steps (Cook & Stang, 2015 ▸). Such supra­molecules are designed by careful selection of the conformational flexibility of the linker groups in multidentate ligands, and the coordination preference of transition-metal ions. We have recently studied the self-assemblies of *m*-xylylene- or 2,7-naphthalene­bis­(methyl­ene)-bridged tetra­dentate bis­(pyridyl­triazole) ligands with Cu^II^ ions to give discrete [2 + 2] metallocycles (Pokharel *et al.*, 2013 ▸, 2014 ▸). In a continuation of our work, we became inter­ested in the design of metalloligands, *i.e.*, metal-containing organic linkers, to produce mixed-metal complexes with different topologies.
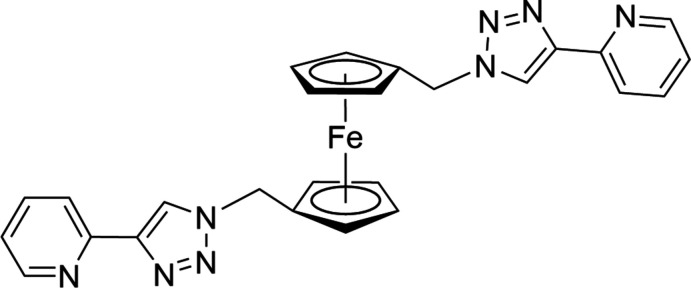


**Figure 1:**
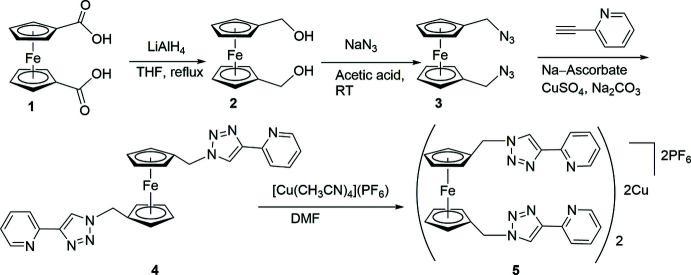
The synthetic scheme showing the formation of the title compound and its complexation with Cu^I^.

Ferrocene, a well-known metallocene, exhibits high thermal stability, reversible electrochemistry, and conformational flexibility, making it an ideal precursor for the development of polymetallic metallo­supra­molecular complexes (Astruc, 2017 ▸). Introduction of the iron(II) center as the structural component of the ligand allows the study of electronic coupling between metal centers in heterometallic metallo­supra­molecular assemblies. Although 1,1′-disubstituted ferrocenes featuring the pyridyl moiety as a donor group have been exploited in metallo­supra­molecular assemblies (Quinodoz *et al.*, 2004 ▸; Buda *et al.*, 1998 ▸; Ion *et al.*, 2002 ▸; Lindner *et al.*, 2003 ▸; Sachsinger & Hall, 1997 ▸), the ferrocene-bridged bis­(pyridyl­triazole)-based tetra­dentate ligands are relatively new in coordination chemistry (Findlay *et al.*, 2018 ▸; Manck *et al.*, 2017 ▸; Romero *et al.*, 2011 ▸). Herein, we report the synthesis of the 1,1′-bis­(methyl­ene­pyridyl­triazole) ferrocene ligand starting from 1,1′-ferrocenedi­carb­oxy­lic acid in a three-step sequence and its complexation with Cu^I^ ions (Fig. 1 ▸).

1,1′-Ferrocenedi­methanol, **2**, was synthesized by reduction of 1,1′-ferrocenedi­carb­oxy­lic acid, **1**, in the presence of LiAlH_4_. The diol was treated with sodium azide in acetic acid following published procedures (Casas-Solvas *et al.*, 2009 ▸) to give 1,1′-bis­(azido­meth­yl)ferrocene, **3** as a viscous liquid. The compound showed a strong IR absorption at 2093 cm^−1^, indicating the formation of the desired diazide (Casas-Solvas *et al.*, 2009 ▸). The diazide was treated with 2-ethynyl­pyridine under ‘click’ conditions (Pokharel *et al.*, 2013 ▸) to give the title compound in 75% yield. This new tetra­dentate ligand based on ferrocene is obtained as an air-stable light-brown crystalline powder.

## Structural commentary   

**Figure 2:**
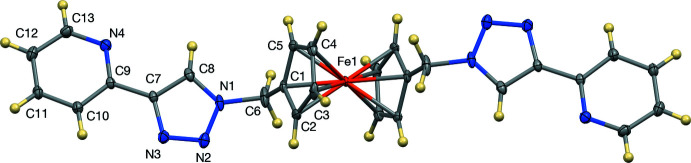
Mol­ecular structure of the title compound showing the atom-numbering scheme. Displacement ellipsoids are drawn at the 50% probability level. Unlabeled atoms are generated by the symmetry operation 1 − *x*, 1 − *y*, 1 − *z*.

The asymmetric unit of the title compound contains one half of the mol­ecule since the Fe^II^ center is on an inversion center, as shown in Fig. 2 ▸. The symmetry in the mol­ecule was also apparent in the NMR data where only one set of signals was found for the protons and carbons of the cyclo­penta­dienyl (Cp) rings, methyl­ene groups, and the pyridyl­triazole units. The Fe—C(Cp) bond lengths are in the range 2.0349 (12)–2.0471 (13) Å [average 2.0498 (13) Å] with the Fe⋯Cp-centroid distance being 1.6550 (6) Å. The Fe—C bond to the substituted carbon [Fe—C1 2.0349 (12)] is shorter than the remaining Fe—C bond lengths, as is seen in similar 1,1′-disubstituted ferrocene derivatives (Glidewell *et al.*, 1994 ▸). The conformation of the ferrocenyl unit is exactly staggered by inversion symmetry, and the centrosymmetry also makes the Cp—Fe—Cp linkage linear and the Cp rings parallel. The C*sp* ^3^ atom, C6, is displaced towards the Fe^II^ center by 0.044 (3) Å from the least-squares plane of the Cp ring. The C_Cp_—C*sp* ^3^ and C_Cp_—N bond lengths involving C6 are 1.4910 (19) and 1.4700 (18) Å, respectively. The pyridyl­triazole moiety is oriented *exo* from the Fe^II^ center, with the least-squares planes of the Cp and triazole rings forming a dihedral angle of 65.68 (5)°. The nitro­gen donor atoms of the pyridyl­triazole units adopt an *anti* conformation, as is often observed in this type of chelating ligand (Crowley & Bandeen, 2010 ▸). The pyridyl and triazole units deviate slightly from coplanarity, with the N3—C7—C9—N4 torsion angle being 167.64 (13)°.

## Supra­molecular features   

**Figure 3:**
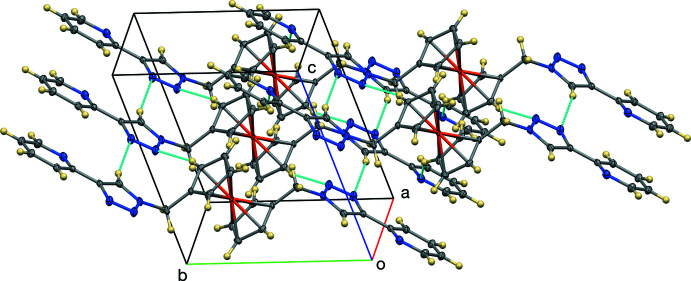
The C—H⋯N network, with displacement ellipsoids at the 50% probability level.

**Figure 4:**
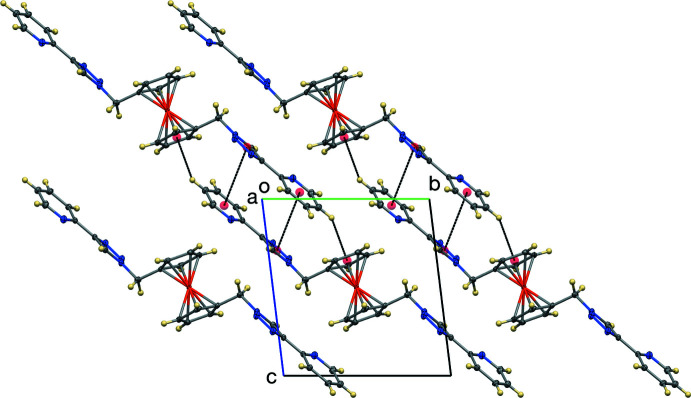
The C—H⋯π and π–π inter­actions, viewed along the *a* axis. Displacement ellipsoids are shown at the 50% probability level.

The crystal structure of the title compound is consolidated by inter­molecular C—H⋯N (Table 1 ▸), C—H⋯π, and π–π inter­actions (Figs. 3 ▸ and 4 ▸). The triazole carbon C8 forms a C—H⋯N inter­action, with a C⋯N distance of 3.601 (2) Å to triazole N3 (at *x* − 1, *y*, *z*) and the Cp carbon atom C5 forms a C—H⋯N inter­action with a C⋯N distance of 3.4240 (19) Å to triazole N2 (at *x* − 1, *y*, *z*). These two contacts form a ring with graph-set motif 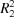
(9) (Etter *et al.*, 1990 ▸). In addition, the triazole carbon C3 forms a C—H⋯N inter­action with a C⋯N distance of 3.4625 (19) Å to pyridyl nitro­gen N4 (at *x*, *y* + 1, *z*). Thus, the C—H⋯N contacts form a two-dimensional network normal to [001]. The pyridyl­triazole moieties stack in an *anti*-parallel fashion about inversion centers. The pyridyl moiety of one mol­ecule has a π–π interaction with the triazole moiety of another mol­ecule with a dihedral angle of 11.27 (10)° and centroid–centroid distance of 3.790 Å (symmetry operation 2 − *x*, −*y*, −*z*). In addition, there are also C—H⋯π inter­actions between the hydrogen atom of the pyridyl moiety with the cyclo­penta­dienyl ring [H12⋯Cp(centroid) = 2.692 Å; symmetry operation 2 − *x*, −*y*, −*z*;]. These two inter­actions thus form centrosymmetric dimers, illustrated in Fig. 4 ▸.

## Database survey   

A search of the Cambridge Structural Database (Version 5.41, update of March 2020; Groom *et al.*, 2016 ▸) for bis­(pyridyl­triazole) with a ferrocene linker gave no results. However, the structure of ferrocene attached to one methyl­ene­pyridyl­triazole, BULQIJ (Crowley *et al.*, 2010 ▸) has been reported. The two pyridyl­triazole units connected with organic linkers, namely *m*-xylylene, VAJVIN (Najar *et al.*, 2010 ▸), and *p*-xylylene as chloro­form solvate, FUJJOK (Crowley & Bandeen, 2010 ▸) have also been reported.

## Complexation with Cu^I^   

**Figure 5:**
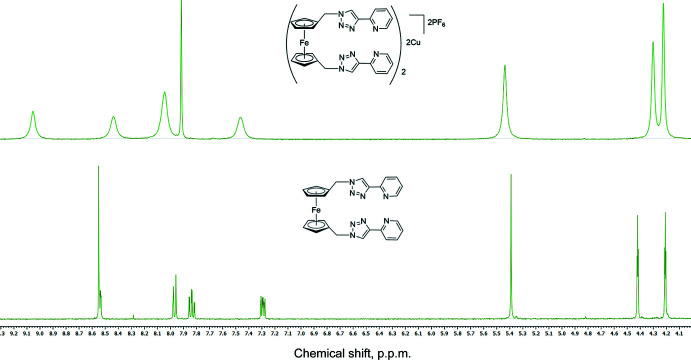
^1^H NMR spectra of ligand **4** (bottom) and its Cu^I^ complex, **5** (top). Both spectra are clipped on the same chemical shift ranges.

**Figure 6:**
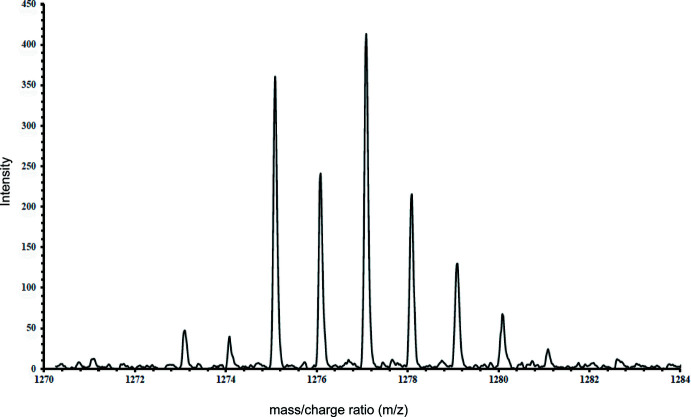
A portion of the high-resolution ESI–MS spectrum of complex **5**

Complexation of the ligand **4** with Cu**^I^** was performed under a nitro­gen atmosphere. When [Cu(CH_3_CN)_4_]PF_6_ was added to a suspension of the ligand in DMF in a 1:1 ratio, the mixture was completely soluble, indicating the formation of a complex. To avoid oxidation of the complex, the resultant solution was diffused with diethyl ether vapor under nitro­gen for 3 d. Under these conditions, a bright-yellow microcrystalline solid was formed. At room temperature, the ^1^H NMR spectrum of the complex showed a simple pattern containing the same set of signals for the ligand, indicative of the presence of one single species in solution. Compared to the spectrum of the free ligand, the proton signals of the complex, especially for the pyridyl­triazole coordination pocket, are shifted downfield (Fig. 5 ▸). Similar retaining of the number of signals and the coupling patterns in the ^1^H NMR spectrum was observed in xylylene-linked bis­(pyridyl­triazole) ligands and their Ag**^I^** complexes (Crowley & Bandeen, 2010 ▸). To further explore the nature of the complex in solution, we examined a MeOH/DMSO solution of the complex by positive-ion electrospray mass spectrometry (ESMS). The ESMS spectrum of the complex contains a peak at 1273.0915 corresponding to [Cu_2_ *L*_2_](PF_6_)^+^ with a similar isotopic pattern as the theoretical simulation (Fig. 6 ▸), indicating the formation of the [2 + 2] complex. Disappointingly, despite obtaining crystalline material, our attempts to obtain crystals suitable for single-crystal X-ray analysis failed.

## Synthesis and crystallization   

**Synthesis of 1,1′-bis­(hy­droxy­meth­yl)ferrocene, 2.** To a stirred solution of 1,1′-ferrocenedi­carb­oxy­lic acid (4.00 g, 14.59 mmol) in dry THF (400.0 mL), 1.0 *M* LiAlH_4_ (58.38 mL, 58.38 mmol) was added dropwise at room temperature under N_2_. The reaction vigorously produced hydrogen gas. The reaction mixture was refluxed for 2 h, by which time the starting compound was consumed, as evidenced by TLC. The reaction was again cooled to room temperature, and ethyl acetate (5 mL) and water (10 mL) were added in sequence with constant stirring. The product was extracted with ethyl acetate (4 × 150 mL). The combined organic layer was dried with anhydrous MgSO_4_ and volatiles removed *in vacuo* to give **2** (3.46 g, 96%) as a brown solid. The analytically pure product was obtained by recrystallization from toluene upon cooling. ^1^H NMR (acetone-*d*_6_, 400 MHz, ppm): δ 4.07 (*t*, 4H, ^3^ *J* = 1.6 Hz, Cp), 4.13 (*t*, 4H, ^3^ *J* = 1.6 Hz, Cp), 4.19 (*t*, 2H, ^3^ *J* = 6.0 Hz, OH), 4.30 (*d*, 4H, ^3^ *J* = 6.0 Hz, C*H*_2_). ^13^C NMR (acetone-*d*_6_, 100 MHz, ppm): δ 67.4, 67.7, 69.6, 89.7.

**Synthesis of 1,1′-bis­(azido­meth­yl)ferrocene, 3.** Caution! Organic azides with low C/N ratio are potentially dangerous. However, we did not encounter any problem during the synthesis of diazide and its subsequent derivatization. To a stirred solution of 1,1′-hy­droxy­methyl­ferrocene (1.50 g, 6.09 mmol) in glacial acetic acid (7.5 mL), sodium azide (2.23 g, 36.5 mmol) was added. The reaction was stirred for 3 h at 323 K under nitro­gen. The reaction mixture was neutralized with a saturated solution of sodium bicarbonate. The product was extracted with chloro­form (2 × 50 mL). The organic phase was dried with anhydrous MgSO_4_ and the volatiles removed *in vacuo* to give **3** (1.50 g, 83%) as a viscous liquid. IR (ATR, cm^−1^): 2092 (*s*), 1733 (*w*), 1240 (*m*). ^1^H NMR (CDCl_3_, 400 MHz, ppm): δ 4.10 (*s*, 4H, C*H*_2_), 4.19 (*t*, ^3^ *J* = 2.0 Hz, Cp), 4.22 (*t*, ^3^ *J* = 2.0 Hz, Cp).

**Synthesis of 1,1′-bis­(pyridyl­triazolylmeth­yl)ferrocene, 4.** To a stirred solution of 1,1′-bis­(azido­meth­yl)ferrocene (1.00 g, 3.34 mmol) in a mixture of DMF and water (4:1) (20 mL), Na_2_CO_3_ (354 mg, 3.34 mmol), CuSO_4_·5H_2_O (333 mg, 1.33 mmol), ascorbic acid (468 mg, 2.66 mmol), and 2-ethynyl­pyridine (862 mg, 8.36 mmol) were added in sequence. The reaction mixture was stirred for 20 h at room temperature, and then poured into an NH_3_/EDTA solution (2.00 g of Na_2_H_2_EDTA·2H_2_O in 5 mL of 28% aqueous NH_3_, diluted to 100 mL with H_2_O) and the mixture extracted with chloro­form (3 x 100 mL). The organic layer was collected, dried over MgSO_4_, and evaporated to dryness. The crude product was purified by trituration with cold diethyl ether to give **4** (1.26 g, 75%) as a light-brown solid. X-ray quality crystals of the compound were obtained by vapor diffusion of diethyl ether into its solution in chloro­form, m.p.: decomposes above 463 K. ^1^H NMR (CDCl_3_, 400 MHz, ppm): δ 4.24 (*t*, 4H, ^3^ *J* = 2.0 Hz, Cp), 4.32 (*t*, 4H, ^3^ *J* = 2.0 Hz, Cp), 5.33 (*s*, 4H, C*H*_2_),7.21(*td*, 2H, ^3^ *J* = 5.2 Hz, ^4^ *J* = 2.0 Hz, Ar), 7.75 (*td*, 2H, ^3^ *J* = 8.0 Hz, ^4^ *J* = 2.0 Hz, Ar), 8.05 (*s*, 2H, triazole-H), 8.15 (*d*, 2H, ^3^ *J* = 7.6 Hz, Ar), 8.53 (*d*, 2H, ^3^ *J* = 4.0 Hz, Ar) ^13^C NMR (CDCl_3_, 100 MHz, ppm): δ 49.8, 69.8, 70.2, 81.9, 120.3, 121.5, 122.9, 137.0, 148.4, 149.4, 150.2. HRESI–MS: *m*/*z* = 501.1416 [**4**+H]^+^ (calculated for C_26_H_23_FeN_8_ 501.1442), 523.1238 [**4**+Na]^+^ (calculated for C_26_H_23_FeN_8_ 523.1261).

**Synthesis of Cu^I^ complex of 1,1′-bis­(pyridyl­triazolylmeth­yl)ferrocene, 5.** To a nitro­gen-purged stirred suspension of **4** (100 mg, 0.20 mmol) in DMF (10 mL), [Cu(CH_3_CN)_4_](PF_6_) (77 mg, 0.20 mmol) was added. The reaction produced a clear yellow solution, which was stirred for 2 h at room temperature. The reaction mixture was diffused with nitro­gen-purged diethyl ether using a cannula for 3 d. The solution was deca­nted and the product was washed with diethyl ether and dried under a slow stream of nitro­gen to give **5** (142 mg, 100%) as a yellow microcrystalline solid. A ^1^H NMR sample was prepared by dissolving the compound in DMSO-*d*_6_ and transferring the solution into an NMR tube under nitro­gen. ^1^H NMR (DMSO-*d*_6_, 400 MHz, ppm): δ 4.21 (*br*, 8H, Cp), 4.29 (*br*, 8H, Cp), 5.43 (*s*, 5.42, 8H, CH_2_, 7.45 (*br*, 4H, Ar), 7.90 (*s*, 4H, triazole-H), 8.04 (*br*, 4H, Ar), 8.43 (*br*, 4H, Ar), 9.05 (*br*, 4H, Ar). HRESI–MS: *m*/*z* = 1273.0915 (Cu_2_ **4**_2_](PF_6_)^+^ (calculated for C_52_H_44_Cu_2_F_6_Fe_2_N_16_P 1273.0914), 563.0650 [Cu**4**](PF_6_)^+^ (calculated for C_26_H_22_CuFeN_8_ 563.0660).

## Refinement   

Crystal data, data collection and structure refinement details are summarized in Table 2 ▸. All H atoms were located in difference maps and then treated as riding in geometrically idealized positions with C—H distances of 0.95 Å (0.99 Å for CH_2_) and with *U*_iso_(H) =1.2*U*_eq_ for the attached C atom.

## Supplementary Material

Crystal structure: contains datablock(s) I, global. DOI: 10.1107/S2056989020011901/pk2645sup1.cif

Structure factors: contains datablock(s) I. DOI: 10.1107/S2056989020011901/pk2645Isup2.hkl

CCDC reference: 2026000

Additional supporting information:  crystallographic information; 3D view; checkCIF report

## Figures and Tables

**Table 1:** Hydrogen-bond geometry (Å, °)

*D*—H⋯*A*	*D*—H	H⋯*A*	*D*⋯*A*	*D*—H⋯*A*
C8—H8⋯N3^i^	0.95	2.68	3.601 (2)	163
C5—H5⋯N2^i^	0.95	2.51	3.4240 (19)	160
C3—H3⋯N4^ii^	0.95	2.73	3.4625 (19)	135
C12—H12⋯Cp_centroid_^iii^	0.95	2.69	3.4861 (15)	142

Symmetry codes: (i) 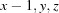; (ii) 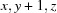; (iii) 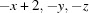.

**Table 2:** Experimental details

Crystal data	
Chemical formula	[Fe(C_13_H_11_N_4_)_2_]
*M*_r_	502.36
Crystal system, space group	Triclinic, *P* 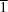
Temperature (K)	90
*a*, *b*, *c* (Å)	5.7905 (3), 9.7461 (5), 10.1720 (4)
α, β, γ (°)	82.064 (3), 84.754 (4), 77.739 (4)
*V* (Å^3^)	554.44 (5)
*Z*	1
Radiation type	Mo *K*α
μ (mm^−1^)	0.71
Crystal size (mm)	0.12 × 0.09 × 0.08

Data collection	
Diffractometer	Bruker Kappa APEXII DUO CCD
Absorption correction	Multi-scan (*SADABS*; Krause *et al.*, 2015 ▸)
*T*_min_, *T*_max_	0.857, 0.945
No. of measured, independent and observed [*I* > 2σ(*I*)] reflections	7058, 4199, 3569
*R*_int_	0.020
(sin θ/λ)_max_ (Å^−1^)	0.769

Refinement	
*R*[*F* ^2^ > 2σ(*F* ^2^)], *wR*(*F* ^2^), *S*	0.041, 0.099, 1.06
No. of reflections	4199
No. of parameters	160
H-atom treatment	H-atom parameters constrained
Δρ_max_, Δρ_min_ (e Å^−3^)	0.98, −0.29

Computer programs: *APEX3* and *SAINT* (Bruker, 2016 ▸), *SHELXS97* (Sheldrick, 2008 ▸), *SHELXL2014/7* (Sheldrick, 2015 ▸), *Mercury* (Macrae *et al.*, 2020 ▸) and *publCIF* (Westrip, 2010 ▸).

## supplementary crystallographic information

### Crystal data

**Table:**

[Fe(C_13_H_11_N_4_)_2_]	*Z* = 1
*M**_r_* = 502.36	*F*(000) = 260
Triclinic, *P*1	*D*_x_ = 1.505 Mg m^−^^3^
*a* = 5.7905 (3) Å	Mo *K*α radiation, λ = 0.71073 Å
*b* = 9.7461 (5) Å	Cell parameters from 2644 reflections
*c* = 10.1720 (4) Å	θ = 3.1–33.1°
α = 82.064 (3)°	µ = 0.71 mm^−^^1^
β = 84.754 (4)°	*T* = 90 K
γ = 77.739 (4)°	Fragment, orange
*V* = 554.44 (5) Å^3^	0.12 × 0.09 × 0.08 mm

### Data collection

**Table:**

Bruker Kappa APEXII DUO CCD diffractometer	4199 independent reflections
Radiation source: fine-focus sealed tube	3569 reflections with *I* > 2σ(*I*)
TRIUMPH curved graphite monochromator	*R*_int_ = 0.020
φ and ω scans	θ_max_ = 33.2°, θ_min_ = 2.0°
Absorption correction: multi-scan (SADABS; Krause *et al.*, 2015)	*h* = −8→8
*T*_min_ = 0.857, *T*_max_ = 0.945	*k* = −14→14
7058 measured reflections	*l* = −15→15

### Refinement

**Table:**

Refinement on *F*^2^	Primary atom site location: structure-invariant direct methods
Least-squares matrix: full	Secondary atom site location: difference Fourier map
*R*[*F*^2^ > 2σ(*F*^2^)] = 0.041	Hydrogen site location: inferred from neighbouring sites
*wR*(*F*^2^) = 0.099	H-atom parameters constrained
*S* = 1.06	*w* = 1/[σ^2^(*F*_o_^2^) + (0.0526*P*)^2^ + 0.140*P*] where *P* = (*F*_o_^2^ + 2*F*_c_^2^)/3
4199 reflections	(Δ/σ)_max_ < 0.001
160 parameters	Δρ_max_ = 0.98 e Å^−^^3^
0 restraints	Δρ_min_ = −0.29 e Å^−^^3^

### Special details

**Table:**

Geometry. All esds (except the esd in the dihedral angle between two l.s. planes) are estimated using the full covariance matrix. The cell esds are taken into account individually in the estimation of esds in distances, angles and torsion angles; correlations between esds in cell parameters are only used when they are defined by crystal symmetry. An approximate (isotropic) treatment of cell esds is used for estimating esds involving l.s. planes.

### Fractional atomic coordinates and isotropic or equivalent isotropic displacement parameters (Å^2^)

**Table:**

	*x*	*y*	*z*	*U*_iso_*/*U*_eq_	
Fe1	0.5000	0.5000	0.5000	0.00859 (7)	
N1	0.9431 (2)	0.11300 (13)	0.36213 (12)	0.0173 (2)	
N2	1.1757 (2)	0.11615 (13)	0.34215 (13)	0.0203 (2)	
N3	1.2663 (2)	0.03069 (13)	0.25245 (13)	0.0175 (2)	
N4	0.9549 (2)	−0.19933 (13)	0.11559 (13)	0.0166 (2)	
C1	0.6904 (2)	0.34473 (13)	0.39618 (12)	0.0123 (2)	
C2	0.7999 (2)	0.46543 (16)	0.37622 (14)	0.0162 (3)	
H2	0.9556	0.4668	0.3976	0.019*	
C3	0.6341 (3)	0.58282 (15)	0.31869 (14)	0.0186 (3)	
H3	0.6595	0.6765	0.2952	0.022*	
C4	0.4251 (3)	0.53559 (15)	0.30257 (13)	0.0169 (3)	
H4	0.2856	0.5923	0.2663	0.020*	
C5	0.4585 (2)	0.38965 (14)	0.34947 (13)	0.0132 (2)	
H5	0.3458	0.3316	0.3498	0.016*	
C6	0.7950 (3)	0.19952 (16)	0.45781 (15)	0.0242 (3)	
H6A	0.8921	0.2067	0.5308	0.029*	
H6B	0.6655	0.1517	0.4970	0.029*	
C7	1.0906 (2)	−0.02744 (13)	0.21606 (13)	0.0125 (2)	
C8	0.8819 (2)	0.02524 (15)	0.28631 (14)	0.0156 (2)	
H8	0.7300	0.0043	0.2820	0.019*	
C9	1.1331 (2)	−0.13233 (13)	0.12183 (13)	0.0119 (2)	
C10	1.3477 (2)	−0.16117 (14)	0.04578 (13)	0.0141 (2)	
H10	1.4713	−0.1132	0.0541	0.017*	
C11	1.3757 (3)	−0.26115 (15)	−0.04177 (14)	0.0166 (3)	
H11	1.5178	−0.2811	−0.0965	0.020*	
C12	1.1936 (3)	−0.33183 (15)	−0.04847 (14)	0.0185 (3)	
H12	1.2090	−0.4018	−0.1069	0.022*	
C13	0.9891 (3)	−0.29783 (16)	0.03193 (15)	0.0187 (3)	
H13	0.8656	−0.3472	0.0277	0.022*	

### Atomic displacement parameters (Å^2^)

**Table:**

	*U*^11^	*U*^22^	*U*^33^	*U*^12^	*U*^13^	*U*^23^
Fe1	0.00934 (12)	0.00722 (12)	0.00860 (12)	−0.00031 (8)	0.00181 (8)	−0.00256 (8)
N1	0.0209 (6)	0.0119 (5)	0.0147 (5)	0.0053 (4)	0.0018 (4)	−0.0026 (4)
N2	0.0243 (6)	0.0143 (5)	0.0223 (6)	−0.0015 (5)	0.0000 (5)	−0.0067 (4)
N3	0.0180 (6)	0.0138 (5)	0.0212 (6)	−0.0032 (4)	0.0011 (4)	−0.0060 (4)
N4	0.0155 (5)	0.0146 (5)	0.0198 (5)	−0.0033 (4)	−0.0001 (4)	−0.0029 (4)
C1	0.0141 (5)	0.0101 (5)	0.0108 (5)	0.0027 (4)	0.0007 (4)	−0.0033 (4)
C2	0.0120 (6)	0.0241 (7)	0.0148 (6)	−0.0060 (5)	0.0042 (4)	−0.0096 (5)
C3	0.0287 (7)	0.0127 (6)	0.0140 (6)	−0.0066 (5)	0.0100 (5)	−0.0036 (5)
C4	0.0191 (6)	0.0175 (6)	0.0103 (5)	0.0047 (5)	−0.0009 (5)	−0.0010 (4)
C5	0.0130 (5)	0.0163 (6)	0.0112 (5)	−0.0033 (4)	0.0012 (4)	−0.0052 (4)
C6	0.0351 (8)	0.0164 (6)	0.0128 (6)	0.0116 (6)	0.0029 (6)	−0.0020 (5)
C7	0.0135 (5)	0.0094 (5)	0.0130 (5)	0.0005 (4)	0.0005 (4)	−0.0011 (4)
C8	0.0152 (6)	0.0135 (6)	0.0148 (6)	0.0026 (5)	0.0000 (5)	−0.0001 (4)
C9	0.0134 (5)	0.0089 (5)	0.0120 (5)	−0.0001 (4)	−0.0002 (4)	−0.0005 (4)
C10	0.0149 (6)	0.0127 (6)	0.0141 (5)	−0.0018 (4)	0.0011 (4)	−0.0019 (4)
C11	0.0175 (6)	0.0181 (6)	0.0129 (6)	−0.0003 (5)	0.0019 (5)	−0.0042 (5)
C12	0.0255 (7)	0.0147 (6)	0.0154 (6)	−0.0023 (5)	−0.0018 (5)	−0.0047 (5)
C13	0.0204 (7)	0.0166 (6)	0.0211 (6)	−0.0063 (5)	−0.0026 (5)	−0.0042 (5)

### Geometric parameters (Å, º)

**Table:**

Fe1—C1	2.0349 (12)	C2—C3	1.423 (2)
Fe1—C1^i^	2.0349 (12)	C2—H2	0.9500
Fe1—C2	2.0453 (13)	C3—C4	1.413 (2)
Fe1—C2^i^	2.0453 (13)	C3—H3	0.9500
Fe1—C5	2.0471 (13)	C4—C5	1.4145 (19)
Fe1—C5^i^	2.0471 (13)	C4—H4	0.9500
Fe1—C4	2.0602 (13)	C5—H5	0.9500
Fe1—C4^i^	2.0603 (13)	C6—H6A	0.9900
Fe1—C3	2.0614 (13)	C6—H6B	0.9900
Fe1—C3^i^	2.0614 (13)	C7—C8	1.3837 (18)
N1—C8	1.3466 (19)	C7—C9	1.4646 (18)
N1—N2	1.3497 (19)	C8—H8	0.9500
N1—C6	1.4700 (18)	C9—C10	1.3987 (18)
N2—N3	1.3185 (17)	C10—C11	1.3838 (19)
N3—C7	1.3656 (18)	C10—H10	0.9500
N4—C13	1.3412 (19)	C11—C12	1.388 (2)
N4—C9	1.3435 (18)	C11—H11	0.9500
C1—C5	1.4248 (19)	C12—C13	1.382 (2)
C1—C2	1.4334 (19)	C12—H12	0.9500
C1—C6	1.4910 (19)	C13—H13	0.9500
			
C1—Fe1—C1^i^	180.0	C5—C1—Fe1	70.03 (7)
C1—Fe1—C2	41.13 (5)	C2—C1—Fe1	69.83 (7)
C1^i^—Fe1—C2	138.87 (5)	C6—C1—Fe1	124.11 (9)
C1—Fe1—C2^i^	138.87 (5)	C3—C2—C1	107.90 (12)
C1^i^—Fe1—C2^i^	41.13 (5)	C3—C2—Fe1	70.34 (8)
C2—Fe1—C2^i^	180.0	C1—C2—Fe1	69.04 (7)
C1—Fe1—C5	40.86 (5)	C3—C2—H2	126.0
C1^i^—Fe1—C5	139.14 (5)	C1—C2—H2	126.0
C2—Fe1—C5	68.47 (5)	Fe1—C2—H2	126.1
C2^i^—Fe1—C5	111.53 (5)	C4—C3—C2	108.01 (12)
C1—Fe1—C5^i^	139.14 (5)	C4—C3—Fe1	69.90 (8)
C1^i^—Fe1—C5^i^	40.85 (5)	C2—C3—Fe1	69.12 (8)
C2—Fe1—C5^i^	111.53 (5)	C4—C3—H3	126.0
C2^i^—Fe1—C5^i^	68.47 (5)	C2—C3—H3	126.0
C5—Fe1—C5^i^	180.00 (7)	Fe1—C3—H3	126.5
C1—Fe1—C4	68.35 (5)	C3—C4—C5	108.52 (12)
C1^i^—Fe1—C4	111.65 (5)	C3—C4—Fe1	69.99 (8)
C2—Fe1—C4	67.96 (6)	C5—C4—Fe1	69.36 (7)
C2^i^—Fe1—C4	112.04 (6)	C3—C4—H4	125.7
C5—Fe1—C4	40.29 (6)	C5—C4—H4	125.7
C5^i^—Fe1—C4	139.71 (6)	Fe1—C4—H4	126.5
C1—Fe1—C4^i^	111.65 (5)	C4—C5—C1	108.24 (12)
C1^i^—Fe1—C4^i^	68.35 (5)	C4—C5—Fe1	70.36 (8)
C2—Fe1—C4^i^	112.04 (6)	C1—C5—Fe1	69.11 (7)
C2^i^—Fe1—C4^i^	67.96 (6)	C4—C5—H5	125.9
C5—Fe1—C4^i^	139.71 (6)	C1—C5—H5	125.9
C5^i^—Fe1—C4^i^	40.29 (5)	Fe1—C5—H5	126.2
C4—Fe1—C4^i^	180.00 (8)	N1—C6—C1	112.79 (12)
C1—Fe1—C3	68.63 (5)	N1—C6—H6A	109.0
C1^i^—Fe1—C3	111.37 (5)	C1—C6—H6A	109.0
C2—Fe1—C3	40.54 (6)	N1—C6—H6B	109.0
C2^i^—Fe1—C3	139.46 (6)	C1—C6—H6B	109.0
C5—Fe1—C3	67.93 (6)	H6A—C6—H6B	107.8
C5^i^—Fe1—C3	112.07 (6)	N3—C7—C8	108.55 (12)
C4—Fe1—C3	40.11 (6)	N3—C7—C9	122.77 (12)
C4^i^—Fe1—C3	139.89 (6)	C8—C7—C9	128.62 (13)
C1—Fe1—C3^i^	111.37 (5)	N1—C8—C7	104.22 (12)
C1^i^—Fe1—C3^i^	68.63 (5)	N1—C8—H8	127.9
C2—Fe1—C3^i^	139.46 (6)	C7—C8—H8	127.9
C2^i^—Fe1—C3^i^	40.54 (6)	N4—C9—C10	122.94 (12)
C5—Fe1—C3^i^	112.07 (6)	N4—C9—C7	115.54 (12)
C5^i^—Fe1—C3^i^	67.93 (6)	C10—C9—C7	121.50 (12)
C4—Fe1—C3^i^	139.89 (6)	C11—C10—C9	118.52 (13)
C4^i^—Fe1—C3^i^	40.11 (6)	C11—C10—H10	120.7
C3—Fe1—C3^i^	180.0	C9—C10—H10	120.7
C8—N1—N2	111.35 (11)	C10—C11—C12	119.08 (13)
C8—N1—C6	129.20 (14)	C10—C11—H11	120.5
N2—N1—C6	119.45 (13)	C12—C11—H11	120.5
N3—N2—N1	107.30 (12)	C13—C12—C11	118.35 (13)
N2—N3—C7	108.57 (12)	C13—C12—H12	120.8
C13—N4—C9	117.19 (12)	C11—C12—H12	120.8
C5—C1—C2	107.33 (12)	N4—C13—C12	123.89 (14)
C5—C1—C6	125.82 (13)	N4—C13—H13	118.1
C2—C1—C6	126.83 (14)	C12—C13—H13	118.1
			
C8—N1—N2—N3	0.31 (16)	N2—N1—C6—C1	88.89 (17)
C6—N1—N2—N3	−179.79 (12)	C5—C1—C6—N1	96.79 (18)
N1—N2—N3—C7	−0.34 (15)	C2—C1—C6—N1	−85.10 (18)
C5—C1—C2—C3	0.44 (14)	Fe1—C1—C6—N1	−174.35 (11)
C6—C1—C2—C3	−177.96 (12)	N2—N3—C7—C8	0.26 (16)
Fe1—C1—C2—C3	−59.84 (9)	N2—N3—C7—C9	−177.32 (12)
C5—C1—C2—Fe1	60.28 (9)	N2—N1—C8—C7	−0.15 (15)
C6—C1—C2—Fe1	−118.12 (13)	C6—N1—C8—C7	179.97 (13)
C1—C2—C3—C4	−0.26 (15)	N3—C7—C8—N1	−0.06 (15)
Fe1—C2—C3—C4	−59.29 (9)	C9—C7—C8—N1	177.33 (13)
C1—C2—C3—Fe1	59.03 (9)	C13—N4—C9—C10	0.0 (2)
C2—C3—C4—C5	−0.02 (15)	C13—N4—C9—C7	−178.92 (12)
Fe1—C3—C4—C5	−58.82 (9)	N3—C7—C9—N4	167.64 (13)
C2—C3—C4—Fe1	58.81 (9)	C8—C7—C9—N4	−9.4 (2)
C3—C4—C5—C1	0.29 (15)	N3—C7—C9—C10	−11.3 (2)
Fe1—C4—C5—C1	−58.92 (9)	C8—C7—C9—C10	171.60 (13)
C3—C4—C5—Fe1	59.21 (9)	N4—C9—C10—C11	1.4 (2)
C2—C1—C5—C4	−0.45 (14)	C7—C9—C10—C11	−179.75 (12)
C6—C1—C5—C4	177.97 (12)	C9—C10—C11—C12	−1.7 (2)
Fe1—C1—C5—C4	59.70 (9)	C10—C11—C12—C13	0.8 (2)
C2—C1—C5—Fe1	−60.15 (9)	C9—N4—C13—C12	−1.1 (2)
C6—C1—C5—Fe1	118.27 (13)	C11—C12—C13—N4	0.6 (2)
C8—N1—C6—C1	−91.24 (19)		

Symmetry code: (i) −*x*+1, −*y*+1, −*z*+1.

### Hydrogen-bond geometry (Å, º)

**Table:**

*D*—H···*A*	*D*—H	H···*A*	*D*···*A*	*D*—H···*A*
C8—H8···N3^ii^	0.95	2.68	3.601 (2)	163
C5—H5···N2^ii^	0.95	2.51	3.4240 (19)	160
C3—H3···N4^iii^	0.95	2.73	3.4625 (19)	135
C12—H12···Cp_centroid_^iv^	0.95	2.69	3.4861 (15)	142

Symmetry codes: (ii) *x*−1, *y*, *z*; (iii) *x*, *y*+1, *z*; (iv) −*x*+2, −*y*, −*z*.

## References

[bb1] Astruc, D. (2017). *Eur. J. Inorg. Chem.* **2017**, 6–29.

[bb2] Bruker (2016). *APEX2* and *SAINT.* Bruker AXS Inc., Madison, Wisconsin, USA.

[bb3] Buda, M., Moutet, J.-C., Saint-Aman, E., De Cian, A., Fischer, J. & Ziessel, R. (1998). *Inorg. Chem.* **37**, 4146–4148. 10.1021/ic971573o 11670538

[bb4] Casas-Solvas, J. M., Ortiz-Salmerón, E., Giménez-Martínez, J. J., García-Fuentes, L., Capitán-Vallvey, L. F., Santoyo-González, F. & Vargas-Berenguel, A. (2009). *Chem. Eur. J.* **15**, 710–725. 10.1002/chem.200800927 19053085

[bb5] Cook, T. R. & Stang, P. J. (2015). *Chem. Rev.* **115**, 7001–7045. 10.1021/cr5005666 25813093

[bb6] Crowley, J. D. & Bandeen, P. H. (2010). *Dalton Trans.* **39**, 612–623. 10.1039/b911276f 20024000

[bb7] Crowley, J. D., Bandeen, P. H. & Hanton, L. R. (2010). *Polyhedron*, **29**, 70–83.

[bb8] Etter, M. C., MacDonald, J. C. & Bernstein, J. (1990). *Acta Cryst.* B**46**, 256–262. 10.1107/s0108768189012929 2344397

[bb9] Findlay, J. A., McAdam, C. J., Sutton, J. J., Preston, D., Gordon, K. C. & Crowley, J. D. (2018). *Inorg. Chem.* **57**, 3602–3614. 10.1021/acs.inorgchem.7b02503 29381330

[bb10] Glidewell, C., Zakaria, C. M., Ferguson, G. & Gallagher, J. F. (1994). *Acta Cryst.* C**50**, 233–238.

[bb11] Groom, C. R., Bruno, I. J., Lightfoot, M. P. & Ward, S. C. (2016). *Acta Cryst.* B**72**, 171–179. 10.1107/S2052520616003954 PMC4822653 27048719

[bb12] Ion, A., Buda, M., Moutet, J.-C., Saint-Aman, E., Royal, G., Gautier-Luneau, I., Bonin, M. & Ziessel, R. (2002). *Eur. J. Inorg. Chem.* pp. 1357–1366.

[bb13] Krause, L., Herbst-Irmer, R., Sheldrick, G. M. & Stalke, D. (2015). *J. Appl. Cryst.* **48**, 3–10. 10.1107/S1600576714022985 PMC4453166 26089746

[bb14] Lindner, E., Zong, R., Eichele, K., Weisser, U. & Ströbele, M. (2003). *Eur. J. Inorg. Chem.* pp. 705–712.

[bb15] Macrae, C. F., Sovago, I., Cottrell, S. J., Galek, P. T. A., McCabe, P., Pidcock, E., Platings, M., Shields, G. P., Stevens, J. S., Towler, M. & Wood, P. A. (2020). *J. Appl. Cryst.* **53**, 226–235. 10.1107/S1600576719014092 PMC6998782 32047413

[bb16] Manck, S., Röger, M., van der Meer, M. & Sarkar, B. (2017). *Eur. J. Inorg. Chem.* pp. 477–482.

[bb17] Najar, A. M., Tidmarsh, I. S. & Ward, M. D. (2010). *CrystEngComm*, **12**, 3642–3650.

[bb18] Pokharel, U. R., Fronczek, F. R. & Maverick, A. W. (2013). *Dalton Trans.* **42**, 14064–14067. 10.1039/c3dt52208c 23995601

[bb19] Pokharel, U. R., Fronczek, F. R. & Maverick, A. W. (2014). *Nat. Commun.* **5**, 5883. 10.1038/ncomms6883 25522935

[bb20] Quinodoz, B., Labat, G., Stoeckli-Evans, H. & von Zelewsky, A. (2004). *Inorg. Chem.* **43**, 7994–8004. 10.1021/ic049913a 15578837

[bb21] Romero, T., Orenes, R. A., Espinosa, A., Tárraga, A. & Molina, P. (2011). *Inorg. Chem.* **50**, 8214–8224. 10.1021/ic200745q 21830760

[bb22] Sachsinger, N. & Hall, C. D. (1997). *J. Organomet. Chem.* **531**, 61–65.

[bb23] Sheldrick, G. M. (2008). *Acta Cryst.* A**64**, 112–122. 10.1107/S0108767307043930 18156677

[bb24] Sheldrick, G. M. (2015). *Acta Cryst.* C**71**, 3–8.

[bb25] Westrip, S. P. (2010). *J. Appl. Cryst.* **43**, 920–925.

